# In-Situ Biofabrication of Silver Nanoparticles in *Ceiba pentandra* Natural Fiber Using *Entada spiralis* Extract with Their Antibacterial and Catalytic Dye Reduction Properties

**DOI:** 10.3390/nano10061104

**Published:** 2020-06-03

**Authors:** Wan Khaima Azira Wan Mat Khalir, Kamyar Shameli, Seyed Davoud Jazayeri, Nor Azizi Othman, Nurfatehah Wahyuny Che Jusoh, Norazian Mohd Hassan

**Affiliations:** 1 Malaysia-Japan International Institute of Technology, Universiti Teknologi Malaysia, Jalan Sultan Yahya Petra, Kuala Lumpur 54100, Malaysia; wkawmk_2505@yahoo.com (W.K.A.W.M.K.); norazizio.kl@utm.my (N.A.O.); nurfatehah@utm.my (N.W.C.J.); 2 Centre for Virus and Vaccine Research, Sunway University, Bandar Sunway 47500, Malaysia; 3 Department of Pharmaceutical Chemistry, Kulliyyah of Pharmacy, International Islamic University Malaysia, Kuantan 25200, Malaysia; norazianmh@iium.edu.my

**Keywords:** silver nanoparticles, *Entada spiralis*, *Ceiba pentandra*, antibacterial assay, catalytic dye reduction

## Abstract

It is believed of great interest to incorporate silver nanoparticles (Ag-NPs) into stable supported materials using biological methods to control the adverse properties of nanoscale particles. In this study, in-situ biofabrication of Ag-NPs using *Entada spiralis (E. spiralis)* aqueous extract in *Ceiba pentandra* (*C. pentandra*) fiber as supporting material was used in which, the *E. spiralis* extract acted as both reducing and stabilizing agents to incorporate Ag-NPs in the *C. pentandra* fiber. The properties of Ag-NPs incorporated in the *C. pentandra* fiber (*C. pentandra*/Ag-NPs) were characterized using UV-visible spectroscopy (UV-vis), X-ray Diffraction (XRD), Field Emission Transmission Electron Microscope (FETEM), Scanning Electron Microscope (Scanning Electron Microscope (SEM), Energy Dispersive X-ray (EDX), Brunauer-Emmett-Teller (BET), Thermogravimetric (TGA) and Fourier Transform Infrared (FTIR) analyses. The average size of Ag-NPs measured using FETEM image was 4.74 nm spherical in shape. The *C. pentandra*/Ag-NPs was easily separated after application, and could control the release of Ag-NPs to the environment due to its strong attachment in *C. pentandra* fiber. The *C. pentandra*/Ag-NPs exposed good qualitative and quantitative antibacterial activities against *Staphylococcus aureus* (ATCC 25923), *Enterococcus faecalis* (ATCC 29212), *Escherichia coli* (ATCC 25922) and *Proteus vulgaris* (ATCC 33420). The dye catalytic properties of *C. pentandra*/Ag-NPs revealed the dye reduction time in which it was completed within 4 min for 20 mg/L rhodamine B and 20 min for 20 mg/L methylene blue dye, respectively. Based on the results, it is evident that *C. pentandra*/Ag-NPs are potentially promising to be applied in wound healing, textile, wastewater treatment, food packaging, labeling and biomedical fields.

## 1. Introduction

With the strong potential of nanotechnology, various types of nanomaterials as an antibacterial agent with strong antibacterial activity have been widely used in many applications to prevent or control the health hazard of microorganisms. The examples of metal and metal oxide nanoparticles with antibacterial properties are zinc oxide (ZnO-NPs), Copper oxide (CuO-NPs) and Ag-NPs [1]. Among these nanoparticles, Ag-NPs have been greatly used in many applications due to their special properties such as the broad spectrum of antibacterial activity, powerful and safe antibacterial nanoparticles, as well as stable nanoparticle dispersion [2]. Moreover, Ag-NPs exhibit special catalytic, chemical, structural, electronic and optical properties different from bulk materials due to the high surface to volume ratio [3]. Such properties of Ag-NPs allow it to be widely used in medical applications (reduce severe burn and human skin treatment), wound dressing, catheter, scaffold, industrial products (shampoo, toothpaste, soaps, detergent, cosmetic products and shoes), pharmaceutical, textile, catalysis, photography, optoelectronics, biological labeling and also photocatalytic applications [4,5,6]. With the increasing production of Ag-NPs in various applications, there are concerns about their release into the environment. High concentrations of Ag-NPs in the environment and small size of the particle can cause potential adverse effects especially on aquatic organisms and human health. In addition, Ag-NPs easily transform into aggregates and settle due to particle-particle interactions. The aggregation of Ag-NPs affects the bioavailability concentration of Ag-NPs, loss of its properties as well as causing ineffective release of more Ag^+^ ions. Therefore, to achieve better stability, control the size of Ag-NPs, high recovery and minimize the release of Ag-NPs into the environment, Ag-NPs should be loaded on supporting materials. 

Currently, the loading of the metal nanoparticles on the supporting materials such as natural fiber has become an intended subject in the field of nanomaterials. One reason is due to these material properties which are renewable, biodegradable and environmentally friendly. The natural fiber loaded with metal nanoparticles can combine the desirable properties of both nanoscale materials and metal nanoparticles. The examples of natural fiber are flax, hemp, jute, kenaf and sisal [7,8]. The loading of metal nanoparticles on the surface of natural fiber can be based on the electrostatic interactions between the negatively charged of the functional group on the fiber surface and positively charged metal nanoparticles [9]. On the other hand, the natural fiber loaded with Ag-NPs can improve its properties which are mechanical, biocompatibility, optical, electronic and magnetic [10]. However, the ability of the natural fiber to absorb a large amount of moisture makes it more prone to microbial attack under certain conditions of humidity and temperature [11]. Therefore, natural fiber facilitating with the antimicrobial agents can be solved the problem. In this study, *C. pentandra* natural fiber was chosen as supporting material for loading of Ag-NPs and is the first time reported in the literature. Comparing with other supporting materials, *C. pentandra* has advantages such as high cellulose content, biodegradability, non-toxicity, abundant availability, low cost and resistance to the microbial attack due to the hydrophobic properties of fiber. The *C. pentandra* fiber is a local plant and can be found abundantly in Malaysia. This plant belongs to the Bombacaceae family which is a white fine silky, lightweight and strong fiber that surrounds the seeds in the pods of the *C. pentandra* tree. The common use of this fiber is in pillow or mattress products, textile industries, water safety equipment, insulation material and upholstery due to their softness and buoyancy [8]. *C. pentandra* fiber consists of single cell fiber with high cellulose compositions [12]. 

Nowadays, due to the environmental damage and pollution resulting from the various industrial processes, many researchers are interested in developing an environmentally friendly method that minimizes and reduces the use of the toxic chemicals. This is because; the toxic chemicals present in the environment can threaten human health and the ecosystem. The conventional method used to synthesize Ag-NPs has some drawbacks in term of toxicity and stability. Therefore, it is worth considering the high potential of plant extracts as a source of reduction Ag^+^ ions to Ag-NPs with their environmentally friendly nature, simplicity and cost-effectiveness. The ability of the *E. spiralis* stem extract to biosynthesis Ag-NPs in *C. pentandra* fiber should be investigated. The in-situ biofabrication of Ag-NPs in *C. pentandra* fiber using natural reducing and stabilizing agent from *E. spiralis* extract was never done or reported in the existing literature. The purpose of this study is to prepare *C. pentandra* fiber as supporting materials for Ag-NPs via in-situ biofabrication process using *E. spiralis* extract and silver nitrate as a silver precursor and to evaluate their antibacterial and catalytic dye reduction properties. The properties of plant-mediated of Ag-NPs deposited in the *C. pentandra* fiber were characterized using UV-vis, XRD, FETEM, SEM, EDX, BET, TGA and FTIR analyses. Their antibacterial and catalytic dye reduction properties of *C. pentandra*/Ag-NPs also were investigated for the potential application like wound healing, textile, wastewater treatment, food packaging and labeling and biomedical fields.

## 2. Materials and Methods

### 2.1. Plant Materials and Chemicals 

**Figure 1:**
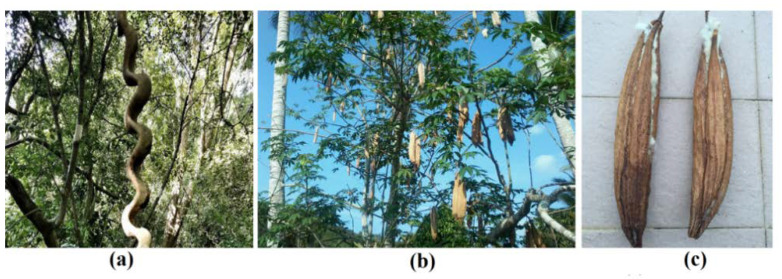
(**a**) *E. spiralis* plant, (**b**) *C. pentandra* plant and (**c**) *C. pentandra* fiber in the seed pod.

The *E. spiralis* stem was collected from the forest in Tasik Chini, Pahang, Malaysia, while the *C. pentandra* fiber was collected during their season from February to April every year from their trees in Besut, Terengganu, Malaysia. The plant of *E. spiralis* and *C. pentandra* are shown in Figure 1. The collected *C. pentandra* fiber was separated from their seed and cleaned with distilled water in order to remove any adhering materials. After being cleaned, the *C. pentandra* fiber was then dried at 60 °C in the oven (Memmert, Germany) overnight. The dried *C. pentandra* fiber was stored in a plastic container for further experiments. Four species of bacteria including two Gram-positive species (*Staphylococcus aureus* (*S. aureus*) (ATCC 25923) and *Enterococcus faecalis* (*E. faecalis*) (ATCC 29212)), as well as two Gram-negative species (*Proteus Vulgaris* (*P. vulgaris*) (ATCC 33420) and *Escherichia coli* (*E. coli*) (ATCC 25922)), were bought from Choice Care Sdn. Bhd, Kuala Lumpur, Malaysia. The silver nitrate (99.85%) was purchased from Acros organic, Geel, Belgium. The sodium hydroxide pellet, nitric acid (69%) and sodium borohydride powder were bought from R&M, Birmingham, UK. The methylene blue dye was bought from (System, Malaysia) and Rhodamine B dye was bought from (R&M, Birmingham, UK). The Mueller Hinton agar (MHA), Mueller Hinton broth (MHB) and gentamicin antibiotic standard (disk) (10 µg) was bought from Difco, Detroit, Michigan, MI, USA. All the chemicals were used without any purification. The deionized water from ELGA Lab-Water/VWS (Buckinghamshire, UK) purification system was used throughout the experiment.

### 2.2. Alkaline Treatment of C. pentandra Fiber

**Figure 2:**
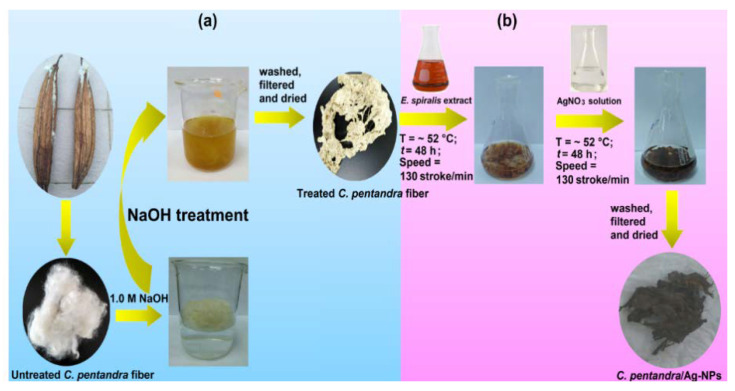
(**a**) Alkaline treatment of *C. pentandra* fiber and (**b**) in-situ biofabrication of Ag-NPs using *E. spiralis* extract and AgNO_3_ solution steps of preparation.

This treatment process was performed using an alkaline solution of sodium hydroxide (NaOH). The mass of 2.0 g of untreated *C. pentandra* fiber and 200 mL of 1.0 M NaOH (1:100) was mixed in the 500 mL beaker as shown in Figure 2a. The mixture was soaked for 5 h at a temperature of ~90 °C. After the soaking process, the NaOH treated *C. pentandra* fiber was washed extensively with distilled water until the pH becomes neutral (pH 7) using pH meter. The washed NaOH treated *C. pentandra* fiber was then dried in an oven at 60 °C overnight. The NaOH treated *C. pentandra* fiber was stored in a plastic container at room temperature until further usage. The NaOH treated *C. pentandra* fiber was referred to as “treated *C. pentandra* fiber” hereafter.

### 2.3. In-Situ Biofabrication of Treated C. pentandra Fiber with Ag-NPs

This in-situ process was done using two steps of the loading process by following the method of Ravindra et al. [11] and Sivaranjana et al. [13] with some modification as shown in Figure 2b. Firstly, the treated *C. pentandra* fiber was immersed in the *E. spiralis* extract. The treated *C. pentandra* fiber was immersed in the 30 mL of *E. spiralis* extract (2.5 g) in a 150 mL conical flask. The *E. spiralis* extract was prepared based on the previous study [14]. The mixture was then shaken at ~52 °C using water bath shaker at 130 strokes/min for 48 h. Then, the fiber was filtered using filter paper and kept for the next step. Secondly, the immersed treated *C. pentandra* fiber in *E. spiralis* extract was then immersed in 3 mL of 0.1 M AgNO_3_ solution as a silver precursor. The mixture was shaken in a water bath shaker at 130 stroke/min and temperature of ~52 °C for 48 h. After the shaking process, the fiber was filtered from the solution and washed with deionized for two times to remove any excess of AgNO_3_ solution before being dried in an oven at 60 °C overnight. The treated *C. pentandra* fiber loaded with Ag-NPs was abbreviated as *C. pentandra*/Ag-NPs hereafter. 

As a control, the treated *C. pentandra* fiber was immersed only in AgNO_3_ solution in the absence of *E. spiralis* extract to determine the significant of *E. spiralis* extract. A mass of 1.0 g of treated *C. pentandra* fiber was immersed into the 30 mL of 0.1 M AgNO_3_ in different conical flasks. The mixture of fiber was shaken in a waterbath shaker (Memmert, Germany) at 130 stroke/min and temperature of 52 °C for 48 h. The fiber was then filtered and washed with 100 mL of deionized water two times to remove any excess of AgNO_3_ solution before dried in the oven overnight at 60 °C. The treated *C. pentandra* fiber immersed only in the AgNO_3_ solution was then stored in the plastic container for further usage and abbreviated as *C. pentandra*/Ag-NO_3_ hereafter. 

### 2.4. Characterization Studies of Untreated C. pentandra Fiber, Treated C. pentandra Fiber and C. pentandra/Ag-NPs 

The UV-vis spectroscopy analysis was started by loading the sample into a solid sample holder. The samples then were scanned from the 300 to 900 nm with a UV-vis spectrophotometer (UV-2600, Shimadzu, Kyoto, Japan) at a medium rate based on transmittance (%) measurement. The XRD analysis was determined using XRD (PANalytical X’pert PRO, Amsterdam, The Netherlands) at 45 kV and a current of 30 mA with Cu-Kα radiation. The fiber sample was cut into small pieces and put on a solid sample holder. The XRD pattern was initiated to scan from 10 to 90° at a 2*θ* angle. The FETEM analysis was determined using FETEM (JEOL, JEM-2100F, Tokyo, Japan). The fiber samples were prepared by sonicating a small piece of fiber in the ethanol solution for 10 min. The samples were then dropped on the copper grate surface using a dropper. The dropped sample was then air-dried completely before running the analysis. The average size of Ag-NPs was measured using image J followed by plotting the histogram of particle size diameter distribution using SPSS software. The particle size distribution histogram is designed based on the counted 100 Ag-NPs incorporated in the *C. pentandra* fiber. The SEM and EDX analyses were observed using Focused Ionized Beam Scanning Electron Microscope (FIBSEM) coupled with EDX using Helios NanoLab G3 UC, FEI, Hillsboro, Oregon, OR, USA. The fiber sample was prepared by placing it on the aluminum holder. The fiber samples were then coated with platinum to increase the electron conductivity of the sample. TGA analysis was studied using a thermogravimetric analyzer (TA Instruments, TGA 55, New castles, Delaware, DE, USA). A known weight of the sample was placed onto platinum crucible and the analysis was carried out under nitrogen flow at the heating rate of 20 °C/min at a temperature from 50 to 700 °C. The surface area of untreated *C. pentandra* fiber and treated *C. pentandra* fiber was determined using surface area analyzer (NOVA touch LX3, Quantachrome Instruments, Palm Beach County, Florida, FL, USA). The analysis was begun by degassing the adsorbents at 50 °C for 180 min at rate 10 °C/min to remove any physisorbed gas. The FTIR analysis was investigated using Attenuated Transmittance Reflectance-Fourier Transform Infrared (ATR-FTIR) spectrometer (Perkin Elmer, Frontier, Waltham, Massachusetts, MA, USA). The small sample of fiber was put on the ATR-FTIR sample holder. The sample was then scanned from 4000 to 650 cm^−1^ wavenumber. 

### 2.5. Silver Content Analysis and Silver Ions Release of C. pentandra/Ag-NPs

The amount of silver (Ag) content in the *C. pentandra*/Ag-NPs was determined using the method proposed by Rehan et al. [15]. The Ag content in *C. pentandra*/Ag-NPs was extracted by immersing 0.2 g dried *C. pentandra*/Ag-NPs with 20 mL of 15% aqueous nitric acid in a conical flask. The mixture was immersed for 2 h in a water bath shaker at 80 °C. After immersed, the filtrate was filtered on Whatman No. 42 filter paper and the Ag^+^ ions concentration was measured using Inductively Coupled Plasma Optical Emissions Spectrophotometer (ICP-OES) (Avio 500, Perkin Elmer, Medtech Park, Singapore) at 328.1 nm wavelength. The Ag content in the *C. pentandra*/fiber sample was calculated using Equation (1):

(1) $$\frac{C\times V}{\left[W\times \left(1-\frac{MC}{1000}\right)\right]}=X$$

where *X* is the Ag content in the fiber (g/kg), *C* is the Ag^+^ ions concentration (mg/L) in extracted solution; *V* is the volume of the extracted solution (L); *W* is the weight of dried *C. pentandra*/Ag-NPs (g); *MC* is the moisture content in dried *C. pentandra*/Ag-NPs (%). The *MC* of *C. pentandra*/Ag-NPs was measured using an oven drying method (method No. 44-15A) as followed by Hussain et al. [16]. The *C. pentandra*/Ag-NPs sample was conditioned at ambient temperature for 24 h. The conditioned sample was then dried in the oven at 105 °C for 2 h. Equation (2) was used to calculate the percentage of moisture content as shown below:

(2) $$\mathrm{Moisture}\;(\%)=\frac{W_{b}-W_{d}}{W_{b}}\times 100$$

where *W_b_* is mass of the sample and *W_d_* is mass of the *C. pentandra*/Ag-NPs after dried in the oven.

The release rate of silver ions from *C. pentandra*/Ag-NPs was investigated by following the method by Zhang et al. [17] and Liu et al. [18]. A mass of 0.05 g of each sample of *C. pentandra*/Ag-NPs at different stirring reaction times (6–144 h) was put into 50 mL of deionized water in the different conical flasks. The mixture was shaken in a water bath shaker at 105 stroke/min and at room temperature. During the shaking time process, 10 mL solution was withdrawn from the release media and another 10 mL of fresh deionized water was added at time intervals of 6, 12, 24, 48, 72 and 96, 120, 144 h. The amount of released Ag^+^ ions was measured using ICP-OES at 328.1 nm wavelength. The procedure was repeated for the sample of *C. pentandra*/Ag-NPs and the results were compared. The experiments were conducted in duplicate and the results were reported in average value. The percentage of Ag^+^ ions release rate was calculated using Equation (3) as below:

(3) $$\mathrm{Ions}\;\mathrm{release}\;\mathrm{rate}\;(\%)=\frac{C_{b}-C_{a}}{C_{b}}\times 100$$

where *C_b_* and *C_a_* are Ag^+^ ions concentration before and after adsorption (mg/L), respectively.

### 2.6. Antibacterial Application

#### 2.6.1. Antibacterial Disk Diffusion Assay

The in-vitro antibacterial activities of *C. pentandra*/Ag-NPs was evaluated qualitatively using the Kirby-Bauer technique [19], which conformed to the recommended standards of Clinical and Laboratory Standards Institute (CLSI). Two Gram-positive bacteria (S. *aureus* and *E. faecalis*), including two Gram-negative bacteria (*E.coli* and *P. vulgaris*) were used in this study. Gentamicin antibiotic disk standard (10 µg), plain disk and *E. spiralis* extract were used as positive and negative controls, respectively. All the glassware, apparatus and culture media were sterilized in an autoclave at 0.1 MPa and 121 °C for 15 min. The agar plate was prepared by solidifying 20 mL of liquid Mueller Hinton agar (MHA) into disposable sterilized petri dishes. The inoculum was prepared by subculturing 100 µL of stock culture bacteria into new sterile Mueller Hinton broth (MHB). The bacterial suspension was incubated overnight at 37 °C in the incubator before adjusted their optical density OD_600_ to 0.10 absorbance (1.5 × 10^6^ CFU/mL) using UV spectrophotometer (Secomam, Champigny sur Marne, France). The different masses of fibers (1, 2 and 4 mg) with the diameter around ~3 mm were applied directly on the MHA plate streaked with adjusted inoculum by following the method by Ravindra et al. [11]. The experiment was carried out in triplicate and the diameter of the inhibition zone was measured after 24 h of incubation at 37 °C. The data were reported in the mean ± standard error of the mean (S.E.) of three experiments.

#### 2.6.2. Percentage of Bacterial Growth Inhibition

The percentage of bacterial growth inhibition in the presence of the *C. pentandra*/Ag-NPs was evaluated quantitatively by following the method of Shameli et al. [20] with some modification. This analysis is based on spectrophotometrically OD value measurements to evaluate bacterial growth. The different masses of fibers (1, 2 and 4 mg) were placed into a sterile 96-well plate. The inoculum was prepared by subculturing 100 µL of stock culture bacteria into new sterile MHB. The inoculum was incubated overnight at 37 °C in the incubator. Afterward, the optical density OD_600_ of the bacterial suspension was adjusted to 1.0 absorbance using UV spectrophotometer (Secomam, Champigny sur Marne, France). This OD_600_ value corresponds to 8 × 10^8^ CFU/mL. The adjusted inoculum was then diluted to 10^5^ (1:1000 dilution) CFU/mL using sterile MHB. The volume of 100 µL of adjusted inoculum (10^5^ CFU/mL) was added to each 96-well plate containing the *C. pentandra*/Ag-NPs. The final volume of each 96-well plate ensures 140 µL by adding sterile MHB. The inoculum of *C. pentandra*/Ag-NPs free medium under the same condition and volume was used as a blank control. The blank control of *C. pentandra*/Ag-NPs sample in MHB media in the absence of inoculum was used. The ampicillin standard (8 µg/mL) was used as positive controls. All the 96-well plates were then sealed using parafilm to avoid the evaporation from each well followed by incubating at 37 °C for 18 h. After the incubation process, the OD_600_ value of the inoculum in each well was determined using a microplate reader (Infinite 200, Tecan, Männedorf, Switzerland) at 600 nm wavelength. The data were reported in the mean ± standard error of the mean (S.E.) of three experiments. The percentage of bacterial growth inhibition rate was calculated using Equation (4) as below:

(4) $$\mathrm{Bacterial}\;\mathrm{growth}\;\mathrm{inhibition}\;(\%)=\left(\frac{OD_{control}-OD_{sample}}{OD_{control}}\right)\times 100$$

where, *OD_control_* is inoculum only, and *OD_sample_* is the differences between *OD_C. pentandra/Ag-NPs_* with bacteria and *OD_C. pentandra/Ag-NPs_* without bacteria (blank).

#### 2.6.3. Statistical Analysis

The statistical analysis was done using ANOVA (one way) analysis to compare the statistical difference among the groups. The analysis was further analyzed the post hoc Tukey HSD test to see which pairs are different between the groups using SPSS version 22. Significant *p* values <0.05 were considered statistically significant.

### 2.7. Catalytic Dye Reduction Application

The catalytic reduction of rhodamine B (RhB) and methylene blue (MB) dye by NaBH_4_ as a model of the reaction has followed a method of Joseph and Mathew [21] and Vidhu and Philip [22] with some modification. Prior to the experiment, a mass of 0.1 g of *C. pentandra*/Ag-NPs was added into 100 mL of dye aqueous solution (20 mg/L). Thereafter, the suspension was magnetically stirred in the dark for 6 h for MB and 1 h for RhB dye, respectively. This step is important to establish the adsorption/desorption equilibrium between dye molecules and the surface of the Ag-NPs. After achieved the equilibrium, the freshly prepared of 10 mL of 0.1 M NaBH_4_ solution was added to start the reaction. An amount of solution was withdrawn every 1 min for RhB dye and every 5 min for MB dye. The solution was then centrifuged and subjected to UV–vis spectrophotometer to measure the absorbance at 554 nm for RhB dye and 664 nm for MB dye. The percentage of dye reduction was calculated using Equation (5) as below:

(5) $$\mathrm{Dye}\;\mathrm{reduction}\;(\%)=\frac{\left(C_{0}-C_{t}\right)}{C_{0}}\times 100$$

where *C*_0_ and *C_t_* are the concentration of the dye solution at a time corresponding to 0 and *t* at the characteristic wavelength, respectively [23].

The kinetics of the dye reduction reaction by *C. pentandra*/Ag-NPs was studied by following the pseudo-first-order and pseudo-second-order kinetic model. The equation of the pseudo-first-order model was described in Equation (6) [21] as below:

(6) $$\ln\left[C_{t}\right]/[C_{0\;}]=kt$$

where, [*C*_0_] is the concentration of dye at time *t* = 0, [*C_t_*] is the concentration at time *t, t* is reaction time and *k* is pseudo-first-order rate constant. The plot of ln[*C_t_*] against *t* was plotted to get the *k* constant value.

Moreover, the equation of the pseudo-second-order model was described in Equation (7) [23] as below:

(7) $$\frac{1}{\;C_{t}}-\frac{1}{C_{0}}=kt$$

where, *C_t_* and *C*_0_ are the concentration of the dye solution at times *t* and 0 min, respectively, *k* is the pseudo-second-order rate constant. The plot of *t/Ct* against *t* was plotted to get the *k* constant value.

## 3. Results and Discussion

### 3.1. Characterization Studies of Alkaline Treated C. pentandra Fiber

**Figure 3:**
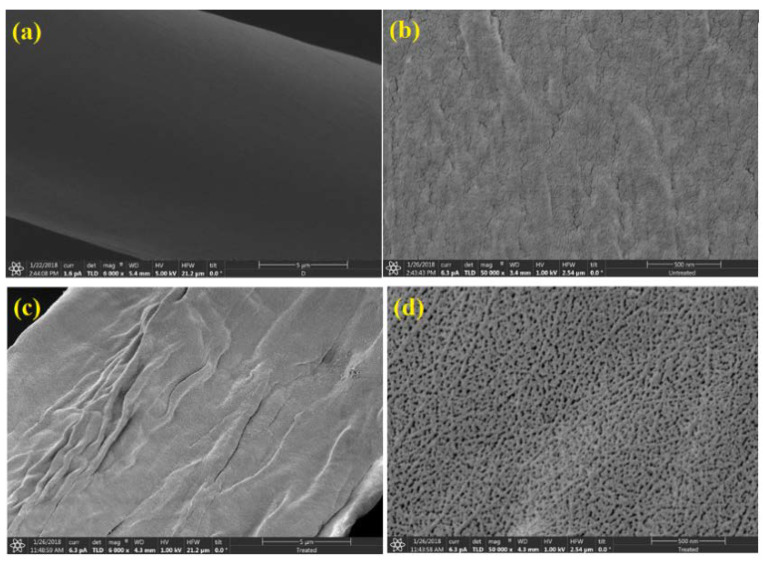
SEM images of (**a**,**b**) untreated *C. pentandra* fiber and (**c**,**d**) treated *C. pentandra* fiber at 6000× and 50,000× magnification, respectively.

The surface morphology of untreated *C. pentandra* fiber was observed using SEM analysis. The smooth surface of untreated *C. pentandra* fiber in the SEM images at different magnification is observed in Figure 3a,b. After alkaline treatment, it clearly showed the treated *C. pentandra* fiber have rougher, more flaky or grooved surface and less attached as seen in Figure 3c,d, respectively. The plant fiber surface becomes rougher and compressed due to the collapse of the lumen structure and removal of the hemicellulose and lignin covered on the cellulose region [24,25]. It is assumed that in the absence of hemicellulose and lignin-containing compositions on the fiber surface enhances the compatibility between fiber and matrix for the deposition of metal nanoparticles [26]. 

**Figure 4:**
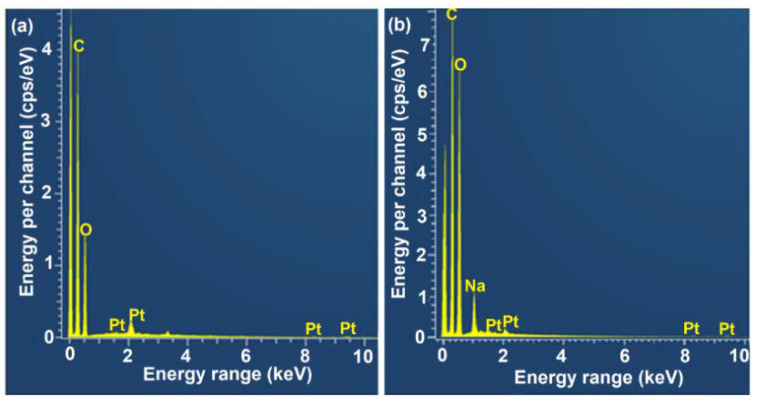
EDX spectra of (**a**) untreated *C. pentandra* fiber and (**b**) treated *C. pentandra* fiber.

The EDX spectra of untreated *C. pentandra* fiber show the major peak of Carbon (C) and Oxygen (O) elements with a percentage of 57.9% and 36.9%, respectively as shown in Figure 4a. These elements are attributed to the hemicellulose and cellulose as the main compositions in the untreated *C. pentandra* fiber. For the EDX spectra of treated *C. pentandra* fiber, besides the C (47.6%) and O (47.4%) as the major element, Na peak appeared at 1.0 KeV with a percentage of 3.8% as shown in Figure 4b. This peak was due to the treatment of *C. pentandra* fiber with NaOH solution. The peak of platinum (Pt) (1.2%) also appeared for both untreated and treated *C. pentandra* fiber due to the fiber was coated with the platinum for the analysis. The percentage of oxygen was increased from 36.9% to 47.4% after alkaline treatment proved that the increased of oxygen density after alkaline treatment. This was due to the disruption of the fiber clusters, removing the hemicellulose and lignin covered on the cellulose region [9,27].

The porosity of the treated *C. pentandra* fiber was further confirmed using surface area analysis based on Langmuir plots. The values of S_L_ surface area obtained for untreated *C. pentandra* fiber was 33.96 m^2^/g. However, after treated with the alkaline solution, the treated *C. pentandra* fiber showed the values of *S*_L_ surface area was increased to 49.65 m^2^/g. This result proved that the porosity of treated *C. pentandra* fiber was increased after treatment and also increased the surface area of *C. pentandra* fiber. The large surface area of treated *C. pentandra* fiber assisted the deposition of Ag-NPs in the fiber surface.

**Figure 5:**
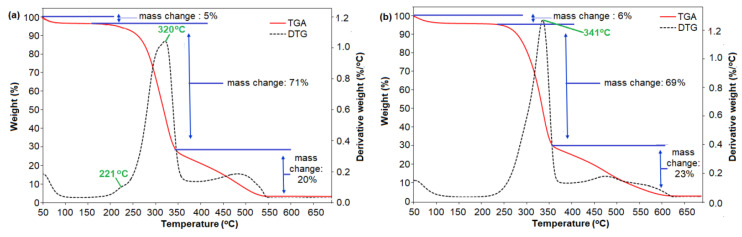
TGA and DTG curves of (**a**) untreated *C. pentandra* fiber and (**b**) treated *C. pentandra* fiber.

The decomposition profile of absorbed water, hemicellulose, cellulose and lignin-containing in *C. pentandra* fiber are depending on their weight compositions. The initial decomposition peak of fibers at a lower temperature (25 to 150 °C) is corresponding to the vaporization of absorbed water in the samples [7,25]. According to [28], hemicellulose starts to decompose at 220–315 °C, while the temperature range of 315–400 °C corresponds to the cellulose decomposition. However, lignin is decomposed at a wider temperature range (200 to 720 °C) [29]. The TGA and derivative thermogravimetry DTG curves of untreated *C. pentandra* fiber and treated *C. pentandra* fiber as a function of temperature are shown in Figure 5. The initial decomposition peak of untreated *C. pentandra* fiber approximately at ~100 °C (5%) is corresponding to vaporization of absorbed water in the samples as shown in Figure 5a. The main *C. pentandra* fiber decomposition occurred at perature around 200–370 °C. This peak region is corresponding to the peak of hemicellulose and lignin [25]. The weight loss of treated *C. pentandra* fiber was slightly decreased than untreated *C. pentandra* fiber from 71% to 69%, respectively. The DTG curve also showed the main *C. pentandra* fiber decomposition occurred in the region from 200–370 °C. The DTG peak was shifted from 320 to 341 °C for untreated and treated *C. pentandra* fiber, respectively corresponding to the cellulose decomposition. This result proved that the base treatment removed the hemicellulose and lignin in the *C. pentandra* fiber and exposed cellulose crystalline region for the attachment of Ag-NPs. The DTG curves of untreated *C. pentandra* fiber also show the small peak of decomposition at 221 °C. This peak is corresponding to the hemicellulose decomposition of untreated *C. pentandra* fiber. This peak is in line with the invisible peak at this region suggesting the removal of hemicellulose of the treated *C. pentandra* fiber during the alkaline treatment. In addition, a more intense peak was observed at 320 °C was due to the decomposition of hemicellulose and lignin. This finding proved that the removal of these compositions during the alkaline treatment to increase the roughness of *C. pentandra* fiber surface and increased the reactive site exposed on the surface for loading of Ag-NPs. The same observation was also reported by Fiore et al. [7], who reported that the removal of hemicellulose from the fiber after treated with NaOH solution. In Figure 5b, the degradation at 390–628 °C show slightly increased to 23% weight loss in comparison with untreated *C. pentandra* fiber corresponding to the increasing number of the macromolecules of *C. pentandra* fiber making it difficult to degrade at a higher temperature only and increased the thermal stability of treated *C. pentandra* fiber. A similar finding was reported by Komal et al. [30] proved that the effective removal of hemicellulose and waxes present on the surface of plant fiber resulted in the enhancement of the thermal stability of plant fiber after alkaline treatment.

### 3.2. In-Situ Biofabrication of Ag-NPs in C. pentandra Fiber(C. pentandra/Ag-NPs)

**Figure 6:**
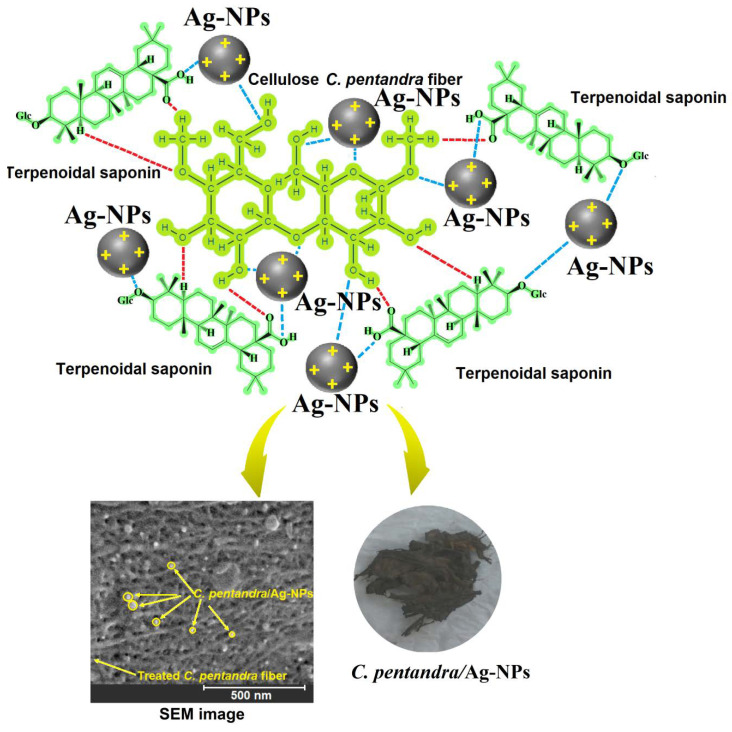
Schematic illustration predicted the formation of in-situ bio-fabrication of Ag-NPs using *E. spiralis* extract in *C. pentandra* fiber.

The success of treated *C. pentandra* fiber incorporated with Ag-NPs can be preliminarily determined visually based on the color of the fiber. The photograph showed that the color of treated *C. pentandra*/Ag-NPs was changed to brown after the bio-fabrication process as shown in Figure 6. The schematic illustration of predicted mechanisms of Ag-NPs got in-situ deposited in the *C. pentandra* fiber also shown in Figure 6. This happened might be due to the interaction of Ag^+^ ions with the functional groups like hydroxyl, carbonyl, ether, aldehyde and acetyl groups that is present in the cellulose of *C. pentandra* fiber and terpenoidal saponin compound of *E. spiralis* extract. The addition of *E. spiralis* extract in the bio-fabrication process help to control the size, shape and stability of Ag-NPs loaded into treated *C. pentandra* fiber. 

### 3.3. Characterization Studies of C. pentandra/Ag-NPs

#### 3.3.1. UV-vis Spectroscopy Analysis

**Figure 7:**
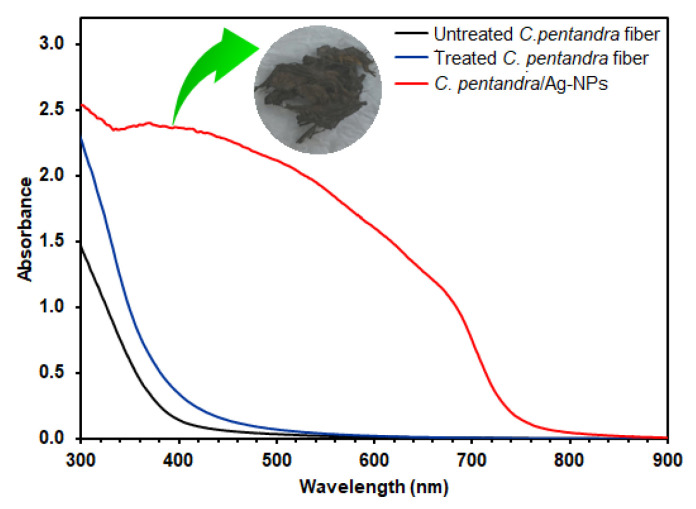
UV–vis spectra of untreated *C. pentandra* fiber, treated *C. pentandra* fiber and *C. pentandra*/Ag-NPs.

The UV-vis spectra of untreated *C. pentandra* fiber, treated *C. pentandra* fiber, *C. pentandra*/Ag-NPs are shown in Figure 7. The Ag peak in UV-vis spectra appeared around 400 to 450 nm. However, no Ag peak was observed in the UV-vis spectra of untreated and treated *C. pentandra* fiber alone. The highest intensity of Ag peak of *C. pentandra*/Ag-NPs appeared around 400–450 nm confirmed the success of Ag-NPs produced in *C. pentandra* fiber in the presence of *E. spiralis* extract [31].

#### 3.3.2. XRD Analysis

**Figure 8:**
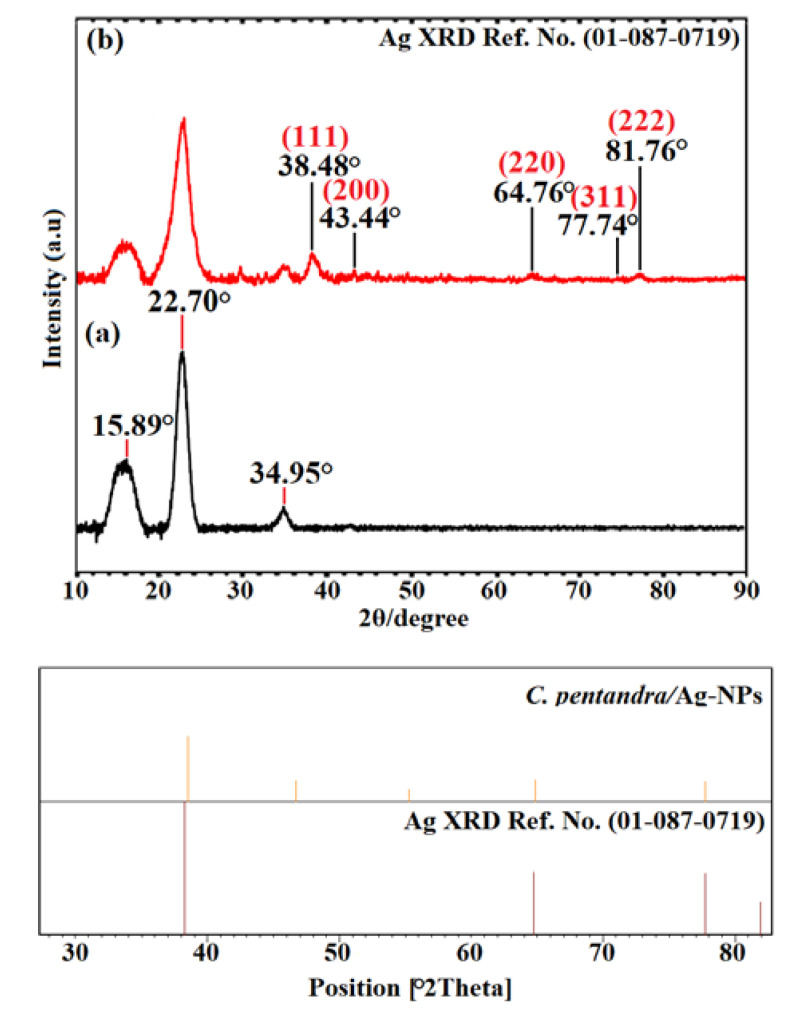
X-ray Diffraction (XRD) patterns of (**a**) treated *C. pentandra* fiber and (**b**) Ag-NPs in *C. pentandra* fiber.

The crystalline structure of Ag-NPs incorporated in *C. pentandra* fiber can be determined using XRD analysis. The XRD pattern of treated *C. pentandra* fiber and *C. pentandra*/Ag-NPs is shown in Figure 8. The peak at 15.89°, 22.70° and 34.95° are corresponding to the amorphous peaks of treated *C. pentandra* fiber as shown in Figure 8a. However, the five diffraction peaks of *C. pentandra*/Ag-NPs appeared at 38.48°, 43.44°, 64.76°, 77.74° and 81.76° of 2*θ* value corresponding to (111), (200), (220), (311) and (222) of indexed of FCC plane of Ag, respectively as shown in Figure 8b. This crystallographic plane is based on ICDD/ICSD X’Pert High Score Plus (Ref. No. 01-087-0719). These peaks show the crystalline structure of Ag-NPs in *C. pentandra* fiber.

#### 3.3.3. FETEM and SAED Pattern Analyses

**Figure 9:**
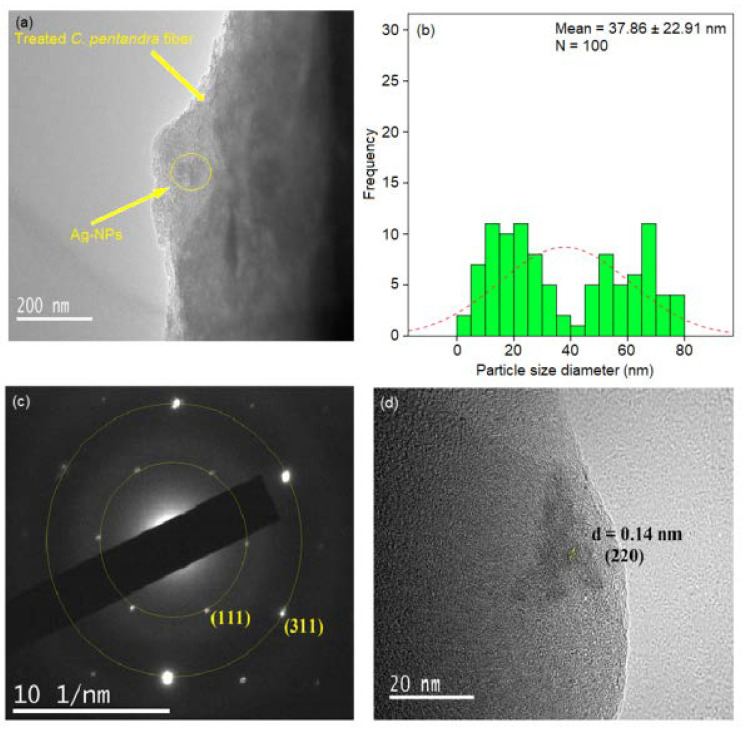
(**a**) Field Emission Transmission Electron Microscope (FETEM) image (**b**) particle size distribution histogram (**c**) SAED pattern and (**d**) lattice d-spacing parameter of *C. pentandra*/AgNO_3_.

The FETEM image of *C. pentandra*/AgNO_3_ is shown in Figure 9a. This result showed that the *C. pentandra* fiber itself also can act as a reducing agent by donating the electron and hydrogen atom from the negatively charged functional groups in the cellulose of fiber. However, the FETEM image observed the big size and aggregation of Ag-NPs were formed. The particle size distribution histogram measured the average size of Ag-NPs incorporated in *C. pentandra* fiber is 37.86 nm as shown in Figure 9b. Figure 9c showed the results of the SAED pattern and lattice d-spacing of *C. pentandra*/AgNO_3_. The two rings around the SAED pattern at (111) and (311) is in line with the FCC plane of Ag-NPs of (111), (200), (220) and (222). Figure 9d showed the image of Ag-NPs with a latticed-spacing of ~0.14 nm correspond to the (220) cubic plane of Ag. 

**Figure 10:**
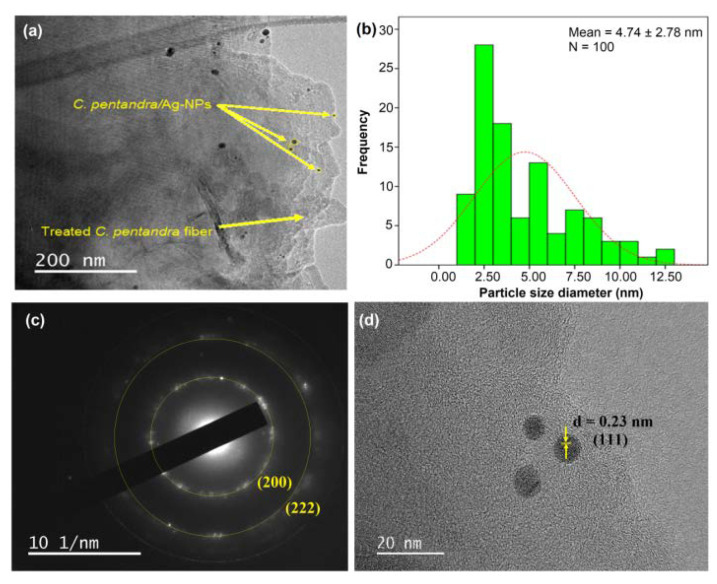
(**a**) FETEM image (**b**) particle size distribution histogram (**c**) SAED pattern and (**d**) latticed-spacing parameter of *C. pentandra*/Ag-NPs.

However, after the addition of *E. spiralis* extract in the process of incorporation of Ag-NPs in *C. pentandra* fiber, the smaller size average size of Ag-NPs was observed as shown in Figure 10a. The shape of Ag-NPs is a spherical shape. The average size of Ag-NPs incorporated in *C. pentandra*/Ag-NPs was decreased to 4.74 nm as shown in Figure 10b. The *E. spiralis* extract was introduced into the *C. pentandra* fiber to act as the reducing agent to reduce Ag^+^ to Ag-NPs and also as stabilizing agent from its negatively charged functional groups in the *E. spiralis* extract. This result proved that the importance and novelty of using *E. spiralis* extract help to control the size, shape and stability of Ag-NPs with a simple technique, nontoxic and no additional of any chemical binding agent. Figure 10c,d showed the results of the SAED pattern and lattice d-spacing of *C. pentandra*/Ag-NPs, respectively. It clearly showed that the two rings around the SAED pattern at (200) and (222) are attributed to the FCC plane of Ag. This pattern is in line with the XRD pattern of the FCC plane of Ag-NPs of (111), (200), (220) and (222) of Ag-NPs. Figure 10c shows the image of *C. pentandra*/Ag-NPs with a latticed-spacing of ~0.23 nm correspond to the (111) FCC plane of Ag. This plane is consistent with the appearance of the highest intensity of XRD peak at angle 38.48° (111) cubic plane of Ag as shown in Figure 8b. 

#### 3.3.4. SEM and EDX Analyses

**Figure 11:**
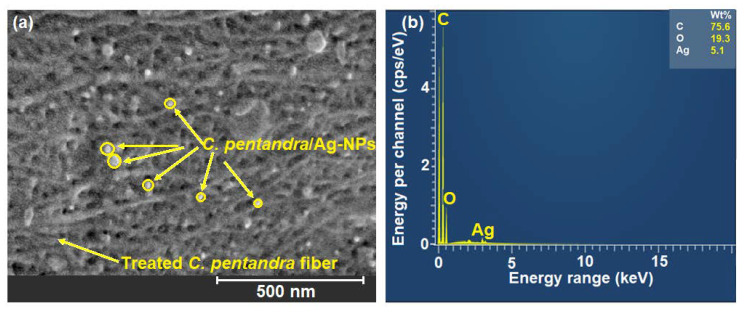
(**a**) SEM image and (**b**) EDX spectra of *C. pentandra*/Ag-NPs.

The SEM image of *C. pentandra*/Ag-NPs is shown in Figure 11a. From the image, it shows that the attachment of *C. pentandra* /Ag-NPs distributed on the rough surface of treated *C. pentandra* fiber. The smaller size of *C. pentandra* /Ag-NPs in a spherical shape surface showing that the significant of *E. spiralis* extract as reducing and stabilizing agent for loading of Ag-NPs in the *C. pentandra* fiber surface. In the EDX spectrum, the Ag peak was detected at 3.0 KeV with a percentage of 5.1% as shown in Figure 11b. The appearance peak of C and O elements with the percentage of 75.6% and 19.3% respectively are corresponding to the cellulose compositions in the treated *C. pentandra* fiber.

#### 3.3.5. TGA Analysis

**Figure 12:**
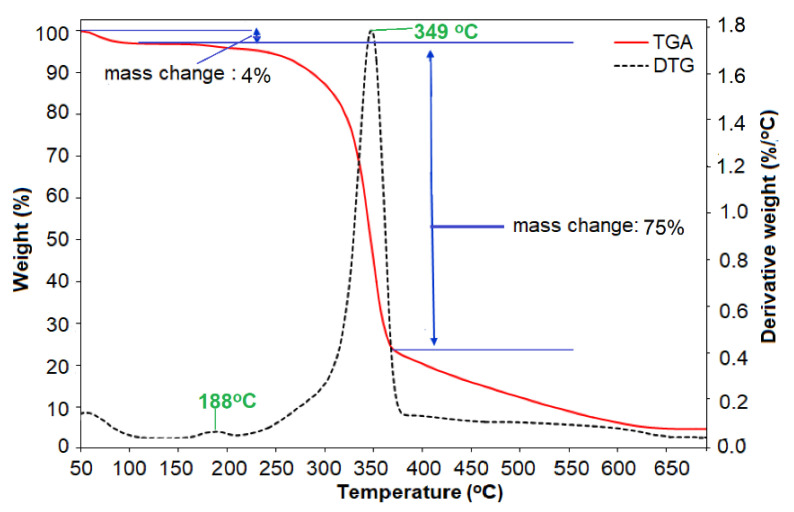
DTG curves of *C. pentandra*/Ag-NPs a function of temperature.

The TGA and DTG curves of *C. pentandra*/Ag-NPs as a function of temperature are shown in Figure 12. The initial peak decomposition of *C. pentandra* /Ag-NPs occurred approximately at ~100 °C (5% weight loss) corresponded to the vaporization of absorbed water in the fiber. The DTG curves show the small peak of *C. pentandra*/Ag-NPs decomposition occurred at 188 °C (1%). This peak corresponded to the peak of NO_3_^−^ that is derived from AgNO_3_ solution. The small peak of ${\mathrm{NO}}_{3}^{-}$ decomposition indicates the Ag^+^ ions were reduced to Ag^0^. The major DTG curve peak decompositions of *C. pentandra*/Ag-NPs occurred at 349 °C (75% of weight loss) corresponding to the cellulose region of the treated *C. pentandra* fiber. This result also shows that the possible mechanism of loading of Ag-NPs into treated *C. pentandra* fiber occurred at the cellulose region. This phenomenon suggests that the Ag-NPs were successfully loaded into the treated *C. pentandra* fiber using AgNO_3_ as Ag precursor. 

#### 3.3.6. FTIR Analysis

**Figure 13:**
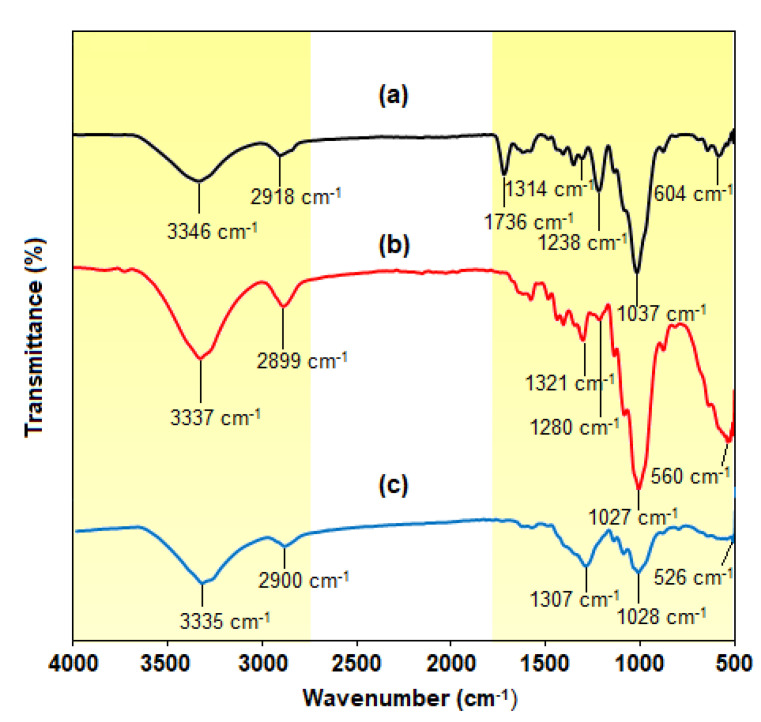
Fourier Transform Infrared (FTIR) spectra of (**a**) untreated *C. pentandra* fiber, (**b**) treated *C. pentandra* fiber and (**c**) *C. pentandra*/Ag-NPs.

The functional groups of cellulose, hemicellulose and lignin in the *C. pentandra* fiber can be analyzed using FTIR analysis. Generally, the cellulose fiber structure consists of carbonyl, carboxyl and aldehyde groups [32]. These compositions untreated and alkaline treated on *C. pentandra* fiber were investigated based on the peak shifting of the related functional groups in the FTIR spectra. The FTIR spectra of untreated *C. pentandra* fiber appeared its absorption bands were at 3346, 2918, 1736, 1314, 1238, 1037 and 604 cm^−1^ as shown in Figure 13a. The peak at 3346 cm^−1^ is responsible for the hydroxyl group stretching of the hydrogen bonding network. At the peak of 2918 cm^−1^ is related to the functional groups of C–H stretching vibration of methyl and methylene groups in cellulose or hemicellulose structure [33]. The peak at 1736 cm^−1^ is corresponding to the C=O stretching vibration of hemicellulose [25]. The peak at 1314 cm^−1^ is corresponding to the C–H bending of aldehyde groups of cellulose structure. The peak at 1238 cm^−1^ is corresponding to the C–O stretching of the acetyl groups in the hemicellulose. The peak at 1037 cm^−1^ is responsible for due to the presence of xylane and the glycosidic linkages of hemicellulose [25]. The peak at 604 cm^−1^ related to the bonding of oxygen from the hydroxyl groups. 

After alkaline treatment of *C. pentandra* fiber, the FTIR spectrum showed the shift in wavenumber or changes in peak intensity explains the types of functional groups involved in the fiber as shown in Figure 13b. The treated *C. pentandra* fiber showed the peak shifted from 3346 to 3337 cm^−1^ suggests the hydroxyl group stretching of the hydrogen bonding network in the treated *C. pentandra* fiber. The peak at 2899 cm^−1^ was shifted from 2918 cm^−1^ for untreated *C. pentandra* fiber suggests the C–H stretching vibration of methyl and methylene groups in the cellulose structure [25]. The peak 1735 cm^−1^ in the untreated *C. pentandra* fiber was disappeared in the treated *C. pentandra* fiber spectra was due to the removal of hemicellulose after alkaline treatment. The peak 1314 cm^−1^ of untreated *C. pentandra* fiber was shifted to 1321 cm^−1^ suggesting C–H bending of aldehyde groups of cellulose structure of treated *C. pentandra* fiber. The intensity of the peak at 1280 cm^−1^ was decreased after alkaline treatment suggesting that the C–O stretching of the acetyl groups. The peak at 1037 cm^−1^ of untreated fiber was shifted to 1027 cm^−1^ with strong peak intensity relate to the presence of xylane and the glycosidic linkages. This major peak shifted can be related to the removal of the hemicellulose structure of treated *C. pentandra* fiber after alkaline treated. This result supported the finding in TGA analysis asserted that the hemicellulose of *C. Pentandra* fiber was removed after alkaline treatment. The peak shifted at 560 cm^−1^ is related to the bonding of oxygen from the hydroxyl groups. 

The FTIR spectrum of *C. pentandra*/Ag-NPs showed the absorption bands at 3335, 2900, 1307, 1028 and 526 cm^−1^ as shown in Figure 13c. After loaded with Ag-NPs, the peak shifted to the 3335 cm^−1^ suggesting to the hydroxyl group stretching of the hydrogen bonding network. The peak shifted to the 2899 cm^−1^ suggesting the C–H stretching vibration of methyl and methylene groups in cellulose and hemicellulose structure. The cellulose structure has an aldehyde group (–CHO) which was oxidized to carboxylic acid, while Ag^+^ ion was reduced to Ag-NPs. This mechanism caused the incorporation of Ag-NPs in *C. pentandra* fiber by strong attachment between Ag-NPs and *C. pentandra* fiber. The peak at 1280 cm^−1^ in the treated *C. pentandra* fiber was disappeared suggesting that the involvement of C–O stretching of the acetyl groups in the hemicellulose for loading Ag-NPs into *C. pentandra* fiber. The peak intensity at 1028 cm^−1^ was decreased after loaded with Ag-NPs suggesting the C–OH of a primary group of the gluco–pyranose ring. The peak shifted at 526 cm^−1^ is related to the bonding of oxygen from the hydroxyl groups in the *C. pentandra* fiber. The FTIR analysis shows the functional groups of *C. pentandra* fiber cellulose structures which consist of carbonyl, carboxyl and aldehyde groups. These functional groups are responsible for the reduction of Ag^+^ ions to Ag-NPs.

### 3.4. Silver Content Analysis in C. pentandra/Ag-NPs

The amount of Ag-NPs deposited in *C. pentandra*/Ag-NPs was determined quantitatively using ICP-OES analysis. The amount of Ag-NPs calculated from *C. pentandra*/Ag-NPs was 40.19 g/kg while for *C. pentandra*/AgNO_3_, the amount of Ag-NPs calculated was only 35.47 g/kg. In the presence of *E. spiralis* extract, increased the reducing agent to the *C. pentandra* fiber for the reduction of Ag^+^ to Ag^0^. Thus, increased the amount of Ag-NPs incorporated in *C. pentandra* fiber. 

### 3.5. Silver Ions Release of C. pentandra/Ag-NPs

**Figure 14:**
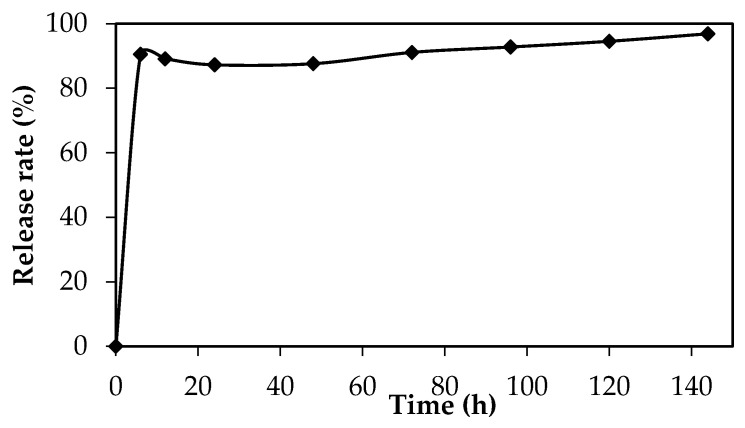
The release rate of Ag^+^ ions from 6 to 144 h of *C. pentandra*/Ag-NPs.

The antibacterial activity of Ag-NPs is related with the release of Ag^+^ ions from Ag-NPs. In this study, the percentage of Ag^+^ ions release rate was observed from 0 to 144 h. In 6 h, the percentage of Ag^+^ ions release rate is 90% and increased to 97% after 144 h as shown in Figure 14. This result supports the potential of *C. pentandra*/Ag-NPs as an antibacterial activity over a long time and highly diffusive of Ag^+^ ions to diffuse in the media and inhibit the bacteria growth. This Ag^+^ ions release caused toxic to the cell of bacteria by generating reactive oxygen species (ROS) and finally caused death of bacteria [34]. Besides that, by supporting Ag-NPs in *C. pentandra* fiber can control the aggregation of the colloidal Ag-NPs which resulted to no bioactivity and bioavailability of Ag-NPs in the media and reduced their antibacterial activity. The usage of Ag-NPs is widely applied due to their slower dissolution rate which led to a continuous release of Ag^+^ ions from Ag-NPs [35]. In addition, the smaller size of Ag-NPs gives advantages by releasing more Ag^+^ ions to the media and inhibits the bacteria growth. The Ag-NPs also are highly diffusive ions into the culture growth medium which are influenced by the oxidation and dilution process from Ag-NPs to Ag^+^ ions [36]. In addition, Ag-NPs also easily dissociate into Ag^+^ ions after contact with water.

### 3.6. Antibacterial Application

#### 3.6.1. Antibacterial Disk Diffusion Assay

**Figure 15:**
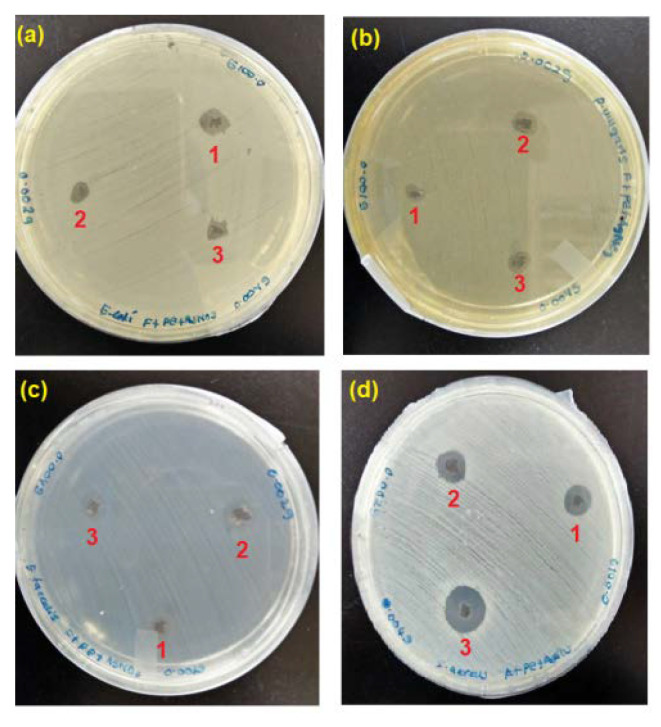
The images of inhibition zones growth against (**a**) *E. coli*, (**b**) *P. vulgaris*, (**c**) *E. faecalis* and (**d**) *S. aureus* around the different mass of *C. pentandra* incorporated with Ag-NPs at 1 (1 mg), 2 (2 mg), and 3 (4 mg).

The qualitative analysis of the antibacterial activity of *C. pentandra*/Ag-NPs was evaluated based on the diameter of growth inhibition zone against the tested bacteria and the results are shown in Figure 15. The *C. pentandra*/Ag-NPs inhibited the growth of all tested bacteria species in a dose-dependent manner. The results showed that the diameter of the growth inhibition zone increased with an increasing amount of *C. pentandra*/Ag-NPs from 1 to 4 mg as shown in Table 1. The significant differences in the diameter of the growth inhibition zone were also observed between the amounts of *C. pentandra*/Ag-NPs used (*p* < 0.05). The multiple comparison post hoc test value showed no significant difference in the diameter of the growth inhibition zone between the mass from 1 to 2 mg of *C. pentandra*/Ag-NPs (*p* > 0.05). By increasing the mass of *C. pentandra*/Ag-NPs up to 4 mg, the diameter of the growth inhibition zone increased significantly (*p* < 0.05). The finding can be explained due to the more Ag-NPs accumulated on the bacterial surface which can enter the cell and damage the nuclei and eventually causing bacterial death [37]. The suppression of bacterial growth increased with the increase in the amount of Ag-NPs is in agreement with the finding from the research by Sowmyya and Lakshmi [38]. 

For the bacteria species, the order of the strongest antibacterial activity of *C. pentandra*/Ag-NPs was *S. aureus.* The less antibacterial activity of *C. pentandra*/Ag-NPs against *P. vulgaris* might be due to the presence of capsule on the bacterial cell wall and the negatively charged of the outer lipid membrane (lipopolysaccharide) cover [39]. The electrostatic repulsion between the nanoparticles and Gram-negative bacteria hinders particles attachment and penetration into the cells [4]. However, the negatively charged of *C. pentandra*/Ag-NPs can bind electrostatically with the negatively charged of teichoic acid present in Gram-positive bacteria cell leading to the enhancement of cell permeability, cytoplasmic leakage and cell death [4,40].

Further analysis was tested on the significant differences in antibacterial activity between Gram-positive and Gram-negative bacteria. Surprisingly, the difference between these bacteria is not significant (*p* > 0.05). This result approved that the *C. pentandra*/Ag-NPs possessed antibacterial activity against both types of bacteria. According to Pollini et al. [41], a diameter of more than 1.0 mm of the microbial growth inhibition zone can be considered as a good antibacterial product. In this study, the *C. pentandra*/Ag-NPs which exhibited more than 1.0 mm of growth inhibition zone has potential antibacterial application, especially in biomedical, textile, wastewater treatment and food packaging areas. The performance of antibacterial activities of *C. pentandra*/Ag-NPs with other Ag-NPs is shown in Table 2. From the Table 2, it showed that the *C. pentandra*/Ag-NPs have comparable antibacterial activities compare to other Ag-NPs. This result showed *C. pentandra*/Ag-NPs have good antibacterial activities for both Gram-positive and Gram-negative bacteria. Yet, the good dispersion of Ag-NPs on the *C. pentandra* surface can contact well with bacteria and releasing more Ag^+^ ions for the effective antibacterial mechanisms.

#### 3.6.2. Percentage of Bacterial Growth Inhibition

The inhibition of bacterial growth was further determined quantitatively based on the OD_600_ value measurements. The percentage of bacterial growth inhibition of *C. pentandra*/Ag-NPs against the tested bacteria is summarized in Table 3. The percentage of bacterial growth inhibition showed high inhibitory activity against all tested bacteria which supported the results obtained by using antibacterial disk diffusion assay analysis. Surprisingly, the percentage of inhibition of bacterial growth by ampicillin antibiotic standard is lower than that of *C. pentandra*/Ag-NPs. However, there is no significant difference in the percentage of inhibition of bacterial growth between the bacteria species used (*p* > 0.05). The percentage of inhibition of bacterial growth of Gram-positive bacteria (*S. aureus* and *E. faecalis*) is lower than Gram-negative bacteria (*E.coli* and *P. vulgaris*). However, there is no significant difference in the percentage of inhibited bacterial growth between the species used (*p* > 0.05). This might be due to the less structural difference in cell wall compositions between Gram-positive and Gram-negative bacteria [43]. The high percentage of bacterial growth inhibition was observed for *C. pentandra*/Ag-NPs at the highest dosage (4 mg) against all tested bacteria (>90%). Conversely, there is no significant difference with the increasing mass of *C. pentandra*/Ag-NPs used from 1 to 4 mg. This can be explained due to the Ag-NPs deposited on the *C. pentandra* fiber surface are randomly distributed on the *C. pentandra* fiber surface. 

### 3.7. Catalytic Dye Reduction Application

#### 3.7.1. Rhodamine B Dye

**Figure 16:**
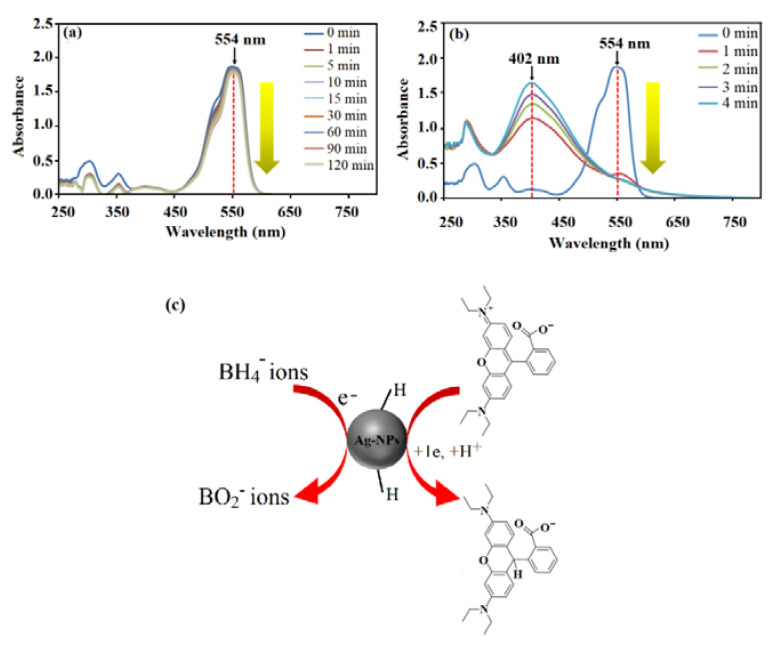
UV–vis spectra of reduction of RhB dye by (**a**) NaBH_4_ alone, (**b**) in the presence of *C. pentandra*/Ag-NPs at 20 mg/L RhB and (**c**) the proposed mechanism of *C. pentandra*/Ag-NPs as nanocatalyst for the reduction of RhB dye.

The catalytic properties of *C. pentandra*/Ag-NPs on the reduction of RhB dye with NaBH_4_ is shown in Figure 16. The decrease in absorbance peak intensity at 554 nm as a function of time reflects the decrease in the concentration of RhB in the system. A controlled experiment was performed on RhB dye in the presence of NaBH_4_ only. The result indicates that in the absence of *C. pentandra*/Ag-NPs, the peak at 554 nm slightly decreased in absorbance of RhB dye solution (20 mg/L) even after 120 min of reaction time (4%) as shown in Figure 16a. This result also displays the reduction of organic dyes by NaBH_4_ is possible but not kinetically favorable due to the kinetic barriers differences in the thermodynamic potential of electron donor (NaBH_4_) and acceptor (RhB dye) [44]. After the addition of *C. pentandra*/Ag-NPs under the same condition, the RhB dye peak–peak at 554 nm decreased quickly and reaches an equilibrium reaction time within 4 min (94%). A new peak was observed around 402 nm corresponding to the peak of Ag as shown in Figure 16b.

This indicates that the ability of *C. pentandra*/Ag-NPs which can act as nanocatalyst and accelerate the reaction. According to Ganguly et al. [45], the reduction of dye molecules generally obeys two stages pathway; initially adsorption of dye molecules onto the Ag-NPs catalyst surface followed by electron transfer phenomenon among catalyst, ${\mathrm{BH}}_{4}^{-}$ ions and dye molecules. The peak observed around 400 to 450 nm at all concentrations of RhB tested corresponding to the peak of Ag as evidence of electron relay process between ${\mathrm{BH}}_{4}^{-}$ ions and RhB dye during the reduction reaction process. This peak was shifted to the blue shift and less intensity than observed in the case of RhB dye reduction. This was due to the fact that the *λ*_max_ of RhB is detached from the SPR absorption of Ag-NPs and reduces the possibility of interaction between these two peaks [21]. The Ag peak was increased with decreasing reduction time until the completed reaction shows that more Ag-NPs involved as electron relay transfer process and RhB is detached during the reduction reaction. 

The proposed mechanism of catalytic reaction by *C. pentandra*/Ag-NPs on the RhB dye reduction can be illustrated in Figure 16c. The redox potential of Ag-NPs is in between the RhB dye (−0.48 V) and NaBH_4_ (−1.33 V) [46]. Thus, can act as electron transfer agents and relay electron from the donor NaBH_4_ to the acceptor RhB dye. Upon the addition of *C. pentandra*/Ag-NPs, the ${\mathrm{BH}}_{4}^{-}$ ions dissociate from NaBH_4_ and donate the electrons as well as transfer it to *C. pentandra*/Ag-NPs. The RhB dye on the surface of *C. pentandra*/Ag-NPs will be accepted that electrons and reduced dye molecules. The negatively charged of *C. pentandra*/Ag-NPs surface brings the RhB dye molecules closer to the surface of *C. pentandra*/Ag-NPs via electrostatic attraction.

#### 3.7.2. Methylene Blue Dye

**Figure 17:**
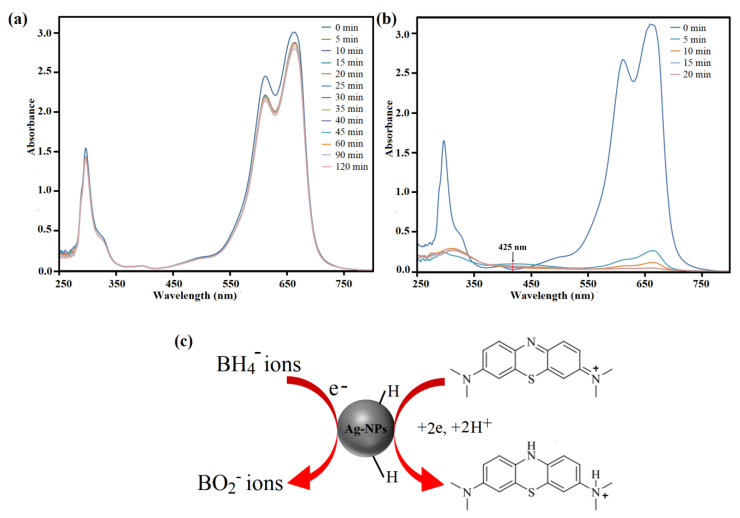
UV–vis spectra of reduction of MB dye by (**a**) NaBH_4_ alone, (**b**) in the presence of *C. pentandra*/Ag-NPs and (**c**) the proposed mechanism of *C. pentandra*/Ag-NPs as nanocatalyst for the reduction of MB dye.

The aqueous solution of MB dye appeared strong absorption peak at 664 nm (*λ*_max_) and reduction of MB dye with time by *C. pentandra*/Ag-NPs was monitored at this peak. The decreasing of absorbance peak at 664 nm with times reflects the decrease in the concentration of MB in the system. This peak was reduced to leucomethylene blue [47]. The reduction of MB dye by NaBH_4_ in the absence of *C. pentandra*/Ag-NPs as nanocatalyst is shown in Figure 17a. From the figure, it shows that a similar trend was observed as in RB dyes where there are no significant changes observed after 120 min. This proved that the reduction of MB dye by NaBH_4_ is not kinetically favorable [48]. However, after the addition of *C. pentandra*/Ag-NPs, the fast reduction time occurred within 20 min (99%) as shown in Figure 17b. The fastest reduction time was achieved at 20 mg/L MB using *C. pentandra*/Ag-NPs due to good distribution of Ag-NPs on the *C. pentandra* fiber surface to be contacted with the MB dye molecule and ${\mathrm{BH}}_{4}^{-}$ ions effectively [45]. The peak observed at 425 nm is corresponding to the peak of Ag. However, a lower intensity of the Ag peak was obtained suggesting that the release of Ag in the solution was controlled by the attachment of Ag-NPs on the *C. pentandra* fiber surface. The amount of Ag detected in all solutions was less than 5%. This result showed that the Ag-NPs loaded in *C. pentandra*/Ag-NPs can limit the release of Ag-NPs in the environment at only low concentrations of Ag. This is happened due to the Ag-NPs after loaded in *C. pentandra* fiber are strongly attached to the *C. pentandra* fiber. This result suggests that the *C. pentandra*/Ag-NPs increase the stability and avoid the agglomeration of Ag-NPs from the attachment on the *C. pentandra* fiber surface without decreasing the catalytic performance of Ag-NPs besides controlling the release of Ag-NPs into the solution. 

The proposed mechanism of catalytic reaction by *C. pentandra*/Ag-NPs on the MB dye reduction can be illustrated in Figure 17c. The catalytic reduction by *C. pentandra*/Ag-NPs followed electron transfer process from Ag-NPs catalyst to MB dye molecules. This electron transfer is depending on the small size of Ag-NPs to make contact with the dye molecules. Then, the MB dye molecules earn the electron from the catalyst surface to reduce its colorless and product. 

#### 3.7.3. Kinetic Study

The kinetic study was studied using a pseudo-first-order and pseudo-second-order model. The applicability of the kinetic models was determined based on the correlation coefficient (R^2^) value of a particular model. The pseudo-first-order kinetic model can be described as the diffusion control process through a boundary [49]. The pseudo-second-order kinetic model is based on the assumption that the rate-determining step is due to chemisorption [50,51]. The pseudo-second-order kinetic process is greatly affected by the number of metal ions on the Ag-NPs [52]. The rate constant (*k*) of the RhB and MB dye reduction process by *C. pentandra*/Ag-NPs can be determined from the slope of the plot for both models as shown in Table 4. Based on the correlation coefficients value (R^2^), the reduction of both RhB and MB dye by *C. pentandra*/Ag-NPs is fitted well with pseudo-first-order model for both RhB and MB dye suggest that the diffusion control process through a boundary was occurred [49]. Diffused ${\mathrm{BH}}_{4}^{-}$ ions produced hydrogen that was attached over the Ag-NPs surface together with dye molecules. Then, the electron transfer between Ag-NPs donated by ${\mathrm{BH}}_{4}^{-}$ ions to the dye molecules were occurred to degrade dye [48].

The performance of *C. pentandra*/Ag-NPs for the catalytic reduction of RhB and MB dye was compared with other reported Ag-NPs catalyst as shown in Table 5. It can be concluded, the reduction rate recorded from this work can be considered as the good nanocatalyst to reduce dyes using an environmentally benign method, simple method, efficient, easy to separate, stable nanoparticles, difficult to swelling and no need to add a binding agent.

## 4. Conclusions

As a conclusion, in-situ bio-fabrication of Ag-NPs in alkaline treated *C. pentandra* fiber as supporting materials were successfully assembled using *E. spiralis* extract and AgNO_3_ solution. This method also is significant to control the bioavailability and bioactivity of Ag-NPs in the antibacterial and dye reduction catalytic application. In addition, the incorporation of Ag-NPs in the *C. pentandra* fiber can control the adverse effect of nanoscale of Ag-NPs by strong association of Ag-NPs with the negatively charged of functional group in the cellulose of *C. pentandra* fiber. The TGA analysis also shows that the possible mechanism of loading of Ag-NPs into treated *C. pentandra* fiber occurred at cellulose region and alkaline treatment helped to expose more cellulose region for loading of Ag-NPs into *C. pentandra* fiber. The characterization studies clearly showed the significant of *E. spiralis* extract as reducing and stabilizing agents added for loading of Ag-NPs on the *C. pentandra* fiber surface. The prepared *C. pentandra*/Ag-NPs also exhibited good antibacterial activity towards both Gram-positive and Gram-negative bacteria *S. aureus, E. faecalis, E. coli* and *P. vulgaris*. *C. pentandra*/Ag-NPs also display the ability as catalytic activity towards the Rhodamine B and methylene blue dyes. The *C. pentandra*/Ag-NPs have potential as a promising nanomaterial for biomedical applications such as for wound healing and coating of biomaterials, wastewater treatment, food packaging and textile in the wide range of microorganism.

## Figures and Tables

**Table 1:** The diameter of the growth inhibition zone of *C. pentandra*/Ag-NPs against different bacteria species.

	The Diameter of Growth Inhibition Zone (mm) ^a^			
	Bacteria Species			
**Sample**	*E. coli*	*P. vulgaris*	*E. faecalis*	*S. aureus*
Mass of *C. pentandra*/Ag-NPs (mg)				
a) 1	7.25 ± 0.20	6.00 ± 0.82	6.75 ± 0.61	8.25 ± 0.20
b) 2	8.00 ± 0.41	6.50 ± 0.41	7.50 ± 1.22	8.75 ± 1.02
c) 4	9.50 ± 0.41	7.50 ± 0.71	8.25 ± 0.20	9.75 ± 0.20
**Control**				
Gentamicin (positive control) (10 µg)	19.50 ± 0.71	23.50 ± 0.41	15.50 ± 0.41	21.75 ± 0.20
*E. spiralis* extract (negative control)	NA	NA	NA	NA
Treated fiber (negative control)	NA	NA	NA	NA
Untreated fiber (negative control)	NA	NA	NA	NA

NA means no activity; ^a^ is mean of the triplicate experiment, ±Standard Error (S.E).

**Table 2:** The comparison on the performance of antibacterial activities based on the zone of growth inhibition by *C. pentandra*/Ag-NPs with other Ag-NPs loaded in supporting materials reported in the literature.

Supporting Material	Bacteria	The Zone of Growth Inhibition (mm)	Ref.
Ag-NPs loaded in *C. pentandra* fiber	*E. coli* *P. vulgaris* *E. faecalis* *S.aurues*	6.5 ± 0.4 4.3 ± 0.7 5.3 ± 0.2 6.8 ± 0.2	This study
Ag-NPs loaded in cotton pad	*E. coli* *L. monocytogens* *S.aureus* *S. epidermis*	6.2 ± 1.4 3.1 ± 1.1 3.3 ± 1.2 3.3 ± 1.2	[1]
ZnO loaded in cotton	*S.aureus* *E. coli*	3.1 ± 0.1 3.3 ± 0.1	[42]
Ag-NPs loaded in Jute fiber	*B. subtillus* *E. coli*	1.0 ± 0.9 2.5 ± 0.8	[33]
Ag-NPs loaded in cotton	*E. coli*	1.5	[11]

**Table 3:** The percentage of bacterial growth inhibition of *C. pentandra*/Ag-NPs against different bacteria species.

	The Percentage of Bacterial Growth Inhibition (%) ^a^			
	Bacteria Species			
**Sample**	*E. coli*	*P. vulgaris*	*E. faecalis*	*S. aureus*
Mass of *C. pentandra*/Ag-NPs (mg)				
a) 1	80.91	93.23	93.87	88.76
b) 2	95.45	93.69	97.96	89.28
c) 4	96.61	97.40	99.60	92.50
Ampicillin (positive control) (8 µg/mL)	81.31	50.27	96.00	97.63

NA means no activity; ^a^ is mean of the triplicate experiment, ±Standard Error (SE).

**Table 4:** The results of pseudo-first-order and pseudo-second-order kinetic model of RhB and MB dye reduction by *C. pentandra*/Ag-NPs.

	Pseudo-First-Order		Pseudo-Second-Order	
Dye	*k* (min^−1^)	R^2^	*k* (min^−1^)	R^2^
RhB	0.7475	0.9912	1.5101	0.8284
MB	0.3582	0.9765	1.8653	0.8767

**Table 5:** Comparison of the catalytic reduction performance of MB and RhB dye by AgNPs nanocatalyst synthesized from another method of preparation.

Method	Catalyst	Dye	Concentration (mg/L)	Reduction Time (min)	*k* (min^−^^1^)	Ref.
NaBH_4_	Ag-NPs loading on *C. pentandra* fiber	MB RhB	20	10 5	0.75 0.36	This study
NaBH_4_	Ag-NPs	MB RhB	0.001	12 10	0.18 0.22	[38]
NaBH_4_	Ag-NPs loading on polypyrrole coated	MB RhB	1.5	10 10	- -	[53]
NaBH_4_	Ag-NPs	MB	5	30	-	[54]
Photocatalytic	Ag-NPs	MB	5	60	0.007	[55]
Photocatalytic	Ag-NPs	MB	15	80	-	[56]
